# Endometrial injury and rFSH before frozen-thawed embryo transfer: a case report

**DOI:** 10.5935/1518-0557.20220066

**Published:** 2023

**Authors:** Emilio Blum-Panchana, Nicolas Gallardo-Molina, Bernardo Blum-Pinto, Medardo Blum-Narváez, Xavier Blum-Rojas, Tatiana Puga-Torres

**Affiliations:** 1 lnnaifest Assisted Reproduction Center, Guayaquil, Ecuador; 2 Medical School, Universidad Espiritu Santo (UEES), Samborondon, Ecuador

**Keywords:** endometrial scratch, rFSH, frozen-thawed embryo transfer

## Abstract

Hormonal treatment as endometrial preparation for frozen-thawed embryo transfer (FET) is routinely carried out with oral, transdermal or combined estradiol supplementation; however, in some cases, there is no optimal endometrial development with this type of stimulation. In this case report, our patient failed to respond to conventional endometrial preparation techniques. For this reason, two unconventional techniques were combined to improve endometrial receptivity; endometrial injury, followed by rFSH administration. As a result of this combination, we achieved endometrium thickness, reaching 8.9 mm on day 15 of the cycle, carrying out the embryo transfer of two blastocysts on day-17 of the cycle, achieving clinical pregnancy and carrying it to completion with the birth of a baby.

## INTRODUCTION

Endometrial preparation for FET plays an important role in *in vitro* fertilization treatments; which normally consists of spontaneous monitoring or hormonal stimulation of the endometrium (Liu *et al*., 2019). In hormone replacement therapy cycles (HRT), endometrial proliferation and follicular growth suppression is achieved by estrogen supplementation; while in the natural cycle, monitoring is carried out without any pharmacological supplement (Sheikhi *et al*., 2018).

Hormonal treatment is routinely carried out with oral, transdermal or combined estradiol supplementation, starting from day 1-3 of the menstrual cycle (Neykova *et al*., 2022); however, in some cases, there is no optimal endometrial development with this type of stimulation. In these specific cases, different approaches have been tried to improve endometrial receptivity, such as endometrial scratch (injury), sildenafil medication or recombinant follicle stimulating hormone (rFSH) (Wafa *et al*., 2021).

In this case report, our patient failed to respond to conventional endometrial preparation techniques, for this reason two unconventional techniques were combined to improve endometrial receptivity.

## CASE DESCRIPTION

A twenty-nine-year-old patient with polycystic ovarian syndrome (PCOS) and secondary infertility (tubal factor) undergoes *in vitro* fertilization treatment with delayed embryo transfer for ovarian hyperstimulation.

The tubal factor, verified by hysterosalpingography and hysteroscopy, was due to a surgical history of endometrial injury due to missed abortion, ovarian cystectomy with right partial oophorectomy, and right salpingectomy due to ectopic pregnancy.

**Figure 1:**
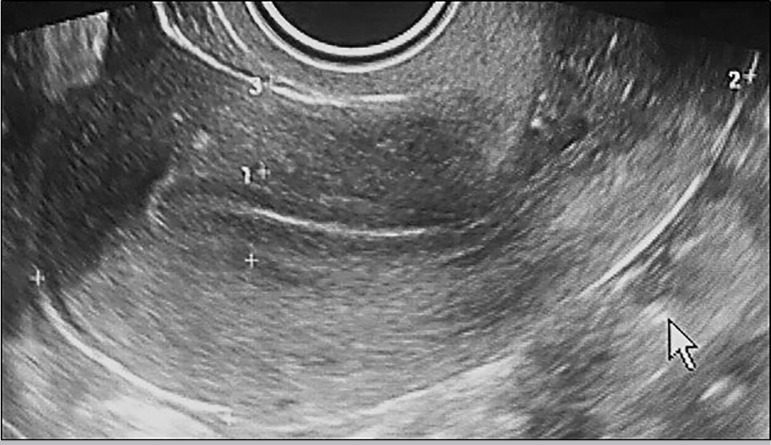
Endometrium measurement on day-15 of the clycle (two days prior to FET) 1. Endometrial thickness, 2. Longitudinal diameter, 3. Anterior-posterior diameter.

**Figure 2:**
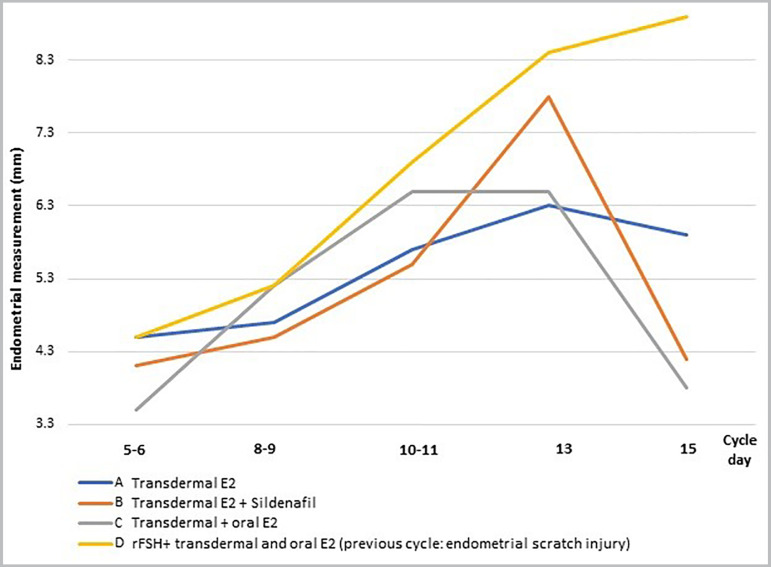
Endometrial development in the different HRT cycles for FET preparation.

Endometrial stimulation protocol for FET was performed, but after different HRT protocols (Figure 1) and one spontaneous cycle monitoring without an adequate response, scratch (endometrial injury) was performed before a new HRT cycle. The subsequently HRT consisted in 75UI rFSH for 13 days beginning day-two of the cycle; transdermal and oral estradiol started on the eighth day. For luteal phase, vaginal progesterone was applied since day thirteen. Endometrial monitoring was performed by transvaginal sonography (Figure 2). The embryo transfer of two blastocysts was performed on day-17 of the cycle.

A quantitative human chorionic gonadotropin hormone test was performed twelve days after embryo transfer, with a value of 390 IU, verifying its duplication 48 hours later. Pregnancy was confirmed by transvaginal sonography 22 days after the embryo transfer, observing an embryo sac with a heartbeat.

Routine pregnancy controls did not show developmental alterations in the first two trimesters. At 28 weeks of gestational age, it showed an alteration in the weight growth of the product, raising suspicion of an amniotic fistula, for which an anti-Rh vaccine was administered. Then, at the gestational age of 32, moderate to severe oligohydramnios was evidenced; for this problem, antibiotic therapy and absolute rest were indicated. At 34 weeks of gestational age, an emergency cesarean section was performed, with a female livebirth baby.

## DISCUSSION

Among the causes for female infertility, tubal factor represents 40% of the cases that may occur for different clinical problems (Babu *et al*., 2017; Yan *et al*., 2020; Rantsi *et al*., 2018; Hoenderboom *et al*., 2019). In this case, the patient presented a tubal factor with right oophorectomy for clinical intervention of ectopic pregnancy, salpingectomy and obstruction of the left tube.

The confirmation of left tube obstruction was performed with hysterosalpingography and diagnostic hysteroscopy technique. Laparoscopy with chromopertubation is the gold-standard for the diagnosis of tubal factor; nevertheless, other widely used methods are contrast sonohysterography and hysterosalpingography (Ott *et al*., 2020). Diagnostic hysteroscopy in the evaluation of infertility is relevant for endometrial evaluation; through this procedure, it is also possible to assess tubal factor infertility, which consists in the visualization of a “flow effect” or air bubbles dispersing through the ostium (Hager *et al*., 2019).

Considering the female tubal factor and a male factor of teratozoospermia, the approach in this case was an *in vitro* fertilization treatment with intracytoplasmic sperm injection (ICSI). During this process, ovarian hyperstimulation occurred, so the embryo transfer was deferred. One of the causes that can trigger ovarian hyperstimulation is PCOS (Schirmer *et al*., 2020). This patient had PCOS, which could have triggered ovarian hyperstimulation, despite receiving low-dose ovarian stimulation.

In the following cycles, the endometrium was prepared to carry out the embryo transfer; however, the endometrial responses in different attempts (HRT protocols and one spontaneous cycle monitoring) were insufficient. At the present time, the endometrial preparation protocol has changed according to the type of transfer; nevertheless, the optimal preparation protocol for FET has not yet been determined, although the literature clarifies that the natural cycle could be superior (Mackens *et al*., 2017). In HRT, the endometrial proliferation is achieved through estrogen supplementation; meanwhile, in the natural cycle the monitoring of the menstrual cycle is generally done without any pharmacological intervention before ovulation (Sheikhi *et al*., 2018).

Considering that the patient did not have a good response with conventional endometrial preparation techniques, as an alternative to endometrial preparation, two unconventional techniques were combined to improve endometrial receptivity: endometrial scratch, followed by rFSH administration. The use of both procedures has been reported separately; however, in this case, it was decided to perform both, considering all previously failed attempts at endometrial preparation. As a result of this combination, we achieved a progressive growth of endometrium thickness, reaching 8.9 mm on day 15 of the cycle (Figure 2), carrying out the embryo transfer of two blastocysts on day-17 of the cycle.

## CONCLUSION

An endometrial scratch injury is a simple procedure that could achieve great benefits for endometrial preparation, and rFSH for HRT could be an alternative in cases where no other type of conventional endometrial preparation protocol works. Future studies should focus on comparing pregnancy outcomes between different endometrial preparation protocols for FET cycles.
